# Physicochemical and Functional Properties of Texturized Vegetable Proteins and Cooked Patty Textures: Comprehensive Characterization and Correlation Analysis

**DOI:** 10.3390/foods11172619

**Published:** 2022-08-29

**Authors:** Shan Hong, Yanting Shen, Yonghui Li

**Affiliations:** Department of Grain Science and Industry, Kansas State University, Manhattan, KS 66506, USA

**Keywords:** textured vegetable protein, meat analogs, physicochemical properties, rehydration capacity, patty textures

## Abstract

Rising concerns of environment and health from animal-based proteins have driven a massive demand for plant proteins. Textured vegetable protein (TVP) is a plant-protein-based product with fibrous textures serving as a promising meat analog. This study aimed to establish possible correlations between the properties of raw TVPs and the corresponding meatless patties. Twenty-eight commercial TVPs based on different protein types and from different manufacturers were compared in proximate compositions, physicochemical and functional properties, as well as cooking and textural attributes in meatless patties. Significant differences were observed in the compositions and properties of the raw TVPs (*p* < 0.05) and were well reflected in the final patties. Of all the TVP attributes, rehydration capacity (RHC) was the most dominant factor affecting cooking loss (r = 0.679) and textures of hardness (r = −0.791), shear force (r = −0.621) and compressed juiciness (r = 0.812) in meatless patties, as evidenced by the significant correlations (*p* < 0.01). The current study may advance the knowledge for TVP-based meat development.

## 1. Introduction

Recently, a massive demand for plant-protein-based diets has been appeared in consumers’ perceptions, which is driven by the multifaceted pressures of animal protein production (e.g., environment, health, animal welfare and ethics issues) [1,2] as well as the high nutritional values and potential health benefits of plant proteins [3]. Plant proteins have been extensively involved in meat products as partial extenders or full replacements to enhance the properties of meat products or to imitate the meat-like texture and taste, thus expanding meat production [2,4]. Shen et al. incorporated a functionally enhanced pea protein, which was prepared through sequential enzymatic modification with protein glutaminase and conjugation with guar gum (pea-glutaminase-guar gum, namely PGG) in beef patties and found that the inclusion of 5% PGG effectively improved the cooking yield while decreasing hardness. The extended beef patty with softer and more tender texture may serve as a good option for elderly people [5].

Textured vegetable protein (TVP) is a processed plant product produced via texturization with fibrous textures, closely imitating the animal muscle meat [6]. TVPs have been derived from several grain proteins, with soy protein and wheat gluten being two of the most primary protein sources [7]. Soy protein has the advantages of its excellent nutritional attributes and highly similar appearance to meat [8]. However, the presence of allergenic protein and genetic modifications narrows down the application of soy protein [9]. Owing to the ability to form anisotropic meat-like structure, wheat gluten is popularly utilized in TVP manufacturing [10]. Nevertheless, the potential of allergy induction and the imbalanced composition of essential amino acids limit the popularity of wheat gluten [10]. By comparison, as a thriving alternative, pea protein is superior in hypoallergenic and non-genetic modification [9]. Other plant proteins receiving increasing attention include chickpea protein, mung bean protein and peanut protein [11,12,13]. Mixed proteins are also processed to offset the imbalance of amino acids [1]. Different TVPs have been widely documented in meat products. For instance, Hidayat et al. studied the effect of TVP on the quality of beef sausages and found that different degrees of TVP substitution improved the water-holding capacity and cooking yield of beef sausages while maintaining a good sensory acceptance up to 30% of TVP replacement [14]. Previously, the integration of TVP in beef patties modified the hardness, cohesiveness and toughness [15,16].

One of the main challenges confronted by meat analogs is the texture. The quality of TVP can be highly affected by the sources and properties of raw materials. The physicochemical and functional properties of plant proteins that are important for texturization include protein solubility, emulsification, gelling ability, water and oil absorption capacity, among others [6,11]. Meanwhile, variations in the texturization process also make significant contributions to the final products. Different technologies have been explored, and some are now available to create the fibrous structures from plant-based proteins, such as fiber spinning, electrospinning, mechanical elongation method, shear cell technology and extrusion [7], of which, extrusion is the dominant approach. During extrusion, taking the low-moisture texturization as an example, moistened proteins are plasticized in the extruder barrel by the application of pressure, heat and mechanical shear. The plasticized mass is then pushed through the die openings during which the mass moisture partially evaporates, and the protein molecules align rapidly to generate fibrous textures [17]. The native structures of proteins are altered in response to the extrusion energy, leading to denaturation and conformation changes along with modifications in physicochemical and functional properties [6,11]. Therefore, manipulating the various extrusion conditions (moisture, temperature, pressure and shear) enables the fabrication of different TVPs with versatile structures and textures. Samard et al. found that TVPs manufactured at 50% moisture content and 130 °C die temperature possessed higher water absorption capacity and superior textural properties in terms of springiness, cohesiveness, chewiness and hardness compared with their counterparts produced at other conditions (40% moisture content and 150 °C die temperature) [18].

Despite the vast information on TVP production and application, little research has looked into the complete physicochemical and functional profiles of TVPs other than the native proteins and focused on how the properties of raw TVPs could be carried over into the end meat-like products. The hypothesis of this study was that the properties of TVP-based meat analogs were closely associated with and determined by the various physical and functional properties of the raw TVPs, and specific correlations existed between the properties of TVPs before and after formulating to patties. Thus, the objective of this research was to provide a comprehensive study on the physicochemical and functional properties of 28 commercial TVPs that are sourced from different protein types, then to evaluate the cooking and textural properties after formulating to the meatless patties, and finally, to establish the potential correlations between the upstream and downstream properties. The systematic study of a relatively high number of samples in the current research may help bridge the gaps between TVP properties and textures in the final plant-based meats, serving as a baseline knowledge to develop desirable plant-based meat analogs for the food industry.

## 2. Materials and Methods

### 2.1. Materials

A total of 28 textured vegetable proteins (labeled as 1–28), with samples 1–14 produced from soy protein, samples 15–21 from pea protein, samples 22–25 from wheat gluten, sample 26 from chickpea protein, sample 27 from pea/chickpea protein mixture and sample 28 from pea/navy bean protein mixture, were obtained from Amazon (Seattle, WA, USA) or other commercial sources. The sample selection mostly depended on the availability of TVP types. Methylcellulose and beetroot powder were purchased from Amazon. Coconut oil and canola oil were purchased from a local grocery store. Other chemicals of analytical grade were obtained from Fisher Scientific (Waltham, MA, USA) unless otherwise stated.

### 2.2. Proximate Composition of TVP 

TVP samples were ground into a fine powder using a coffee mill for 30 s. The protein content of TVP powder was determined following the combustion method (AACC Method 46-30.01) using a LECO analyzer with the nitrogen to protein conversion factor of 6.25. Moisture content (AACC Method 44-19.01) was measured as the weight loss of approximately 2 g of each powder that was dried at 135 °C for 2 h in an automatic oven (Isotemp Oven, Fisher Scientific, Waltham, MA, USA). Ash content (AACC method 08-01.01) was determined by incinerating around 3 g of sample powder in a furnace (Fisher Scientific, Waltham, MA, USA) at 575 °C overnight. The measurement of fat content was modified from a previous method [19]. Briefly, 2 g of each TVP powder was mixed with 30 mL ethyl ether with continuous shaking for 30 min at 250 rpm (Orbital Shaker Model 361, Fisher Scientific, Waltham, MA, USA). After centrifugation for 10 min at 10,000× *g* (Benchmark Hermle Z 366 K centrifuge, Hermle Labortechnik GmbH, Wehingen, Germany), the supernatant was collected in an aluminum dish pan and allowed to evaporate overnight in a fume hood to obtain the extracted fat. All the proximate compositions were analyzed in triplicate. Total carbohydrate content was determined by subtracting the total contents of protein, fat, ash and moisture from 100 percent, as in the following equation:

Total carbohydrate (%) = 100 − (protein + moisture + ash + fat) (%)(1)

### 2.3. Protein Solubility 

Protein solubility was determined as previously reported with slight modifications [20]. The ground sample (1.5 g) was suspended in 30 mL of 0.5% *w*/*v* KOH followed by shaking for 20 min at 250 rpm and room temperature (RT). The supernatant was decanted after centrifugation at 10,000× *g* for 20 min (Benchmark Hermle Z 366 K centrifuge, Hermle Labortechnik GmbH, Wehingen, Germany). The extraction was repeated once, and the precipitate was freeze dried (Labconco FreeZone 4.5 Lite Benchtop FreezeDryer, Labconco Corporation, Kansas City, MO, USA). The lyophilized precipitate as the insoluble protein was then subjected to protein content analysis as described in Section 2.2. The protein solubility was calculated as the percentage of soluble protein to the total protein. The measurements were conducted in triplicate.

(2) $$\mathrm{Protein}\;\mathrm{Solubility}\;(\%)=100-\frac{\mathrm{wt}\;\mathrm{of}\;\mathrm{precipitate}\;\left(g\right)\times \;\mathrm{protein}\;\mathrm{content}\;\mathrm{in}\;\mathrm{precipitate}\;\left(\%\right)}{\mathrm{wt}\;\mathrm{of}\;\mathrm{TVP}\;\mathrm{powder}\;\left(g\right)\times \;\mathrm{protein}\;\mathrm{content}\;\mathrm{in}\;\mathrm{TVP}\;\left(\%\right)}\times 100$$

### 2.4. Water Absorption Capacity and Oil Absorption Capacity 

Water/oil absorption capacity (WAC/OAC) tests were performed following our previous method [5] with minor modifications. For WAC, 0.6 g (W_0_) of ground sample was dispersed in 10 mL deionized (DI) water in a pre-weighed 15 mL centrifuge tube (W_1_). The mixture was vortexed thoroughly and allowed to stand for 5 min at RT. After centrifugation for 30 min at 3000× *g* (Benchmark Hermle Z 366 K centrifuge, Hermle Labortechnik GmbH, Wehingen, Germany), the supernatant was discarded, and the tube with the residue was inverted to stand for 5 min before re-weighing (W_2_). For OAC, 1 g (O_0_) of each sample was mixed thoroughly with 10 mL canola oil in a pre-weighed 15 mL centrifuge tube (O_1_). The mixture was allowed to stand for 30 min at RT before centrifugation at 3000× *g* for 30 min. After discarding the oil, the tube containing the protein sediment was inverted for 10 min to drain the excess oil followed by re-weighing (O_2_). The WAC and OAC were expressed as grams of water and oil absorbed per gram of sample using the following equations, respectively. Each sample was carried out in triplicate.

(3) $$\mathrm{WAC}\;(g\;H_{2}O/g\;\mathrm{sample})=\frac{W_{2}-{\;W}_{1}-{\;W}_{0}}{W_{0}}$$

(4) $$\mathrm{OAC}\;(g\;\mathrm{oil}/g\;\mathrm{sample})=\frac{O_{2}-{\;O}_{1}-{\;O}_{0}}{O_{0}}$$

### 2.5. Viscosity 

The viscosity characteristics of TVPs were measured on a Rapid Visco Analyzer (RVA) (RVA4500, Perten Instruments, Hägersten, Sweden) using the AACC method 76-21.02 (13 min procedure) with slight modifications. Approximately 7.0 g of each ground sample was placed in a canister and mixed with 25 mL DI water. The TVP powder slurry was heated to 50 °C and equilibrated for 1 min, followed by ramping up to 95 °C within 4 min while stirring at 960 rpm for the initial 10 seconds for thorough dispersion and at 160 rpm for the remaining RVA test. After holding for 3 min at 95 °C, the mixture was cooled to the initial 50 °C within 4 min and held for another 2 min. The peak time (the time at which peak viscosity occurred), peak viscosity (the maximum hot paste viscosity) and final viscosity (the viscosity at the end of the test after cooling to 50 °C and holding at this temperature) were recorded. Each sample was analyzed in duplicate. 

### 2.6. Bulk Density 

Dry TVP was filled in a 1 L graduated cylinder with gentle tapping twice to eliminate the interspace of the crumbles. The volume and the weight were recorded, and the bulk density was calculated as the weight per volume (g/L). Two measurements were taken for each sample.

### 2.7. Rehydration Capacity 

Twenty grams of dry TVP was rehydrated in 300 mL DI water (1:15 solid to liquid ratio) for 2 h at room temperature (RT), followed by draining for 1 h on a mesh screen. The final weight was recorded to quantify the rehydration capacity (RHC) as follows. Each sample was conducted in triplicate.

(5) $$\mathrm{RHC}\;(g\;H_{2}O/g\;\mathrm{sample})=\frac{\mathrm{weight}\;\mathrm{after}\;\mathrm{rehydration}\;\left(g\right)-\mathrm{weight}\;\mathrm{before}\;\mathrm{rehydration}\;\left(g\right)}{\mathrm{weight}\;\mathrm{before}\;\mathrm{rehydration}\;\left(g\right)}$$

### 2.8. Textural Properties of Rehydrated TVP 

The textural properties of rehydrated TVPs were characterized by texture profile analysis (TPA) using a TA-XT Plus texture analyzer (Stable Micro System, Godalming, Surrey, UK) following our previous method [5]. Prior to measurement, dry TVP crumbles were hydrated in DI water at 1:15 mass ratio as described above. Approximately 15 g of each hydrated sample was transferred to a Petri dish for up to 1 cm height. TPA was performed by a two-compression test using a cylinder prober (2-inch diameter) at a strain compression rate of 50% with 20 g trigger force and a pre-test speed of 1.0 mm/s, a post-test speed of 5.0 mm/s and a test speed of 1.0 mm/s. The textural attributes of hardness (the peak force during the first compression), resilience (the ratio of the downstroke area to the upstroke area under the first compression peak), cohesiveness (the area under the first compression curve divided by the area under the second compression curve), springiness (the ratio of the time to reach the peak during the second compression over the time to reach the peak during the first compression) and chewiness (hardness × cohesiveness × springiness) were collected. Each TVP sample was conducted in four replicates.

### 2.9. Preparation of TVP Patties

Prior to formulation, dry TVPs were allowed to hydrate for 2 h followed by draining for 1 h at RT as described in Section 2.7. The drained TVP was ground for 30 s using a food processor (Ninja BL770 Mega Kitchen System, SharkNinja Operating LLC, Needham, MA, USA) to achieve uniform and smaller particles (2–3 mm). Thereafter, 100 g of the hydrated and processed TVP was mixed with 2 g methylcellulose, 1 g NaCl, 1 g beetroot powder and 20 g pre-melted coconut oil by hand thoroughly to obtain a homogeneous mixture. The mixtures of approximately 20 g weight were then formed into patties using a cylindrical mold, following which the patties were placed in a fridge (4 °C) for 30 min to solidify the shape. The patties were grilled on a non-stick plate without adding additional oil until the internal temperature reached 71 °C as measured by a probe thermometer and were allowed to cool for 40 min at RT before further analysis. The patty formulation was optimized and finalized during preliminary experiments and standardized by the authors.

### 2.10. Determination of Cooking Properties

Cooking loss was determined by the percentage weight difference of a patty before and after cooking using the following equation:

(6) $$\mathrm{Cooking}\;\mathrm{loss}\;(\%)=\frac{\mathrm{raw}\;\mathrm{patty}\;\mathrm{weight}\;\left(g\right)-\;\mathrm{cooked}\;\mathrm{patty}\;\mathrm{weight}\;\left(g\right)}{\mathrm{raw}\;\mathrm{patty}\;\mathrm{weight}\;\left(g\right)}\;\times \;100$$

The diameter shrinkage of the patties was determined by random measurement of the diameter at three different locations of the raw and cooked patties and was expressed according to the following equation:

(7) $$\mathrm{Diameter}\;\mathrm{shrinkage}\;(\%)=\frac{\mathrm{raw}\;\mathrm{patty}\;\mathrm{diameter}\;\left(\mathrm{mm}\right)-\;\mathrm{cooked}\;\mathrm{patty}\;\mathrm{diameter}\;\left(\mathrm{mm}\right)}{\mathrm{raw}\;\mathrm{patty}\;\mathrm{diameter}\;\left(\mathrm{mm}\right)}\;\times \;100$$

The moisture content of both raw and cooked patties was measured as described in Section 2.2 by drying 2 g samples at 135 °C for 2 h. The moisture retention was then calculated as below:

(8) $$\mathrm{Moisture}\;\mathrm{retention}\;(\%)=\frac{\mathrm{Cooked}\;\mathrm{patty}\;\mathrm{weight}\;\left(g\right)\times \;\mathrm{moisture}\;\mathrm{in}\;\mathrm{cooked}\;\mathrm{patty}\;\left(\%\right)}{\mathrm{Raw}\;\mathrm{patty}\;\mathrm{weight}\;\left(g\right)\times \;\mathrm{moisture}\;\mathrm{in}\;\mathrm{raw}\;\mathrm{patty}\;\left(\%\right)}\;\times \;100$$

For fat retention, both raw and cooked patties were freeze dried to remove the water, and the fat in lyophilized patties was then extracted and determined, as in Section 2.2. Fat retention was quantified according to the following equation:

(9) $$\mathrm{Fat}\;\mathrm{retention}\;(\%)=\frac{\mathrm{Cooked}\;\mathrm{patty}\;\mathrm{weight}\;\left(g\right)\times \;\mathrm{fat}\;\mathrm{in}\;\mathrm{cooked}\;\mathrm{patty}\;\left(\%\right)}{\mathrm{Raw}\;\mathrm{patty}\;\mathrm{weight}\;\left(g\right)\times \;\mathrm{fat}\;\mathrm{in}\;\mathrm{raw}\;\mathrm{patty}\;\left(\%\right)}\;\times \;100\;$$

All cooking measurements were performed in four replicates per TVP treatment, except for cooking loss. Cooking loss was determined using eight different patties for each TVP.

### 2.11. Textural Property of TVP-Based Patty 

Texture profile analysis of the cooked patties was carried out following the same procedure as in Section 2.8. Four patties from each TVP treatment were assigned for the determination of TPA. 

### 2.12. Shear Force Measurement

The shear force test was performed using the same texture analyzer assembled with a Warner–Bratzler Shear Blade (Stable Micro System, Godalming, Surrey, UK). Cooked patties were cut into 2 cm wide strips (around 1 cm thickness) before being sheared straight through the perpendicular cooked patty surface at a test speed of 5 mm/s. The corresponding force–distance curves were recorded. The shear force value was collected as the maximal peak force of shearing. Each TVP treatment was analyzed in four strip replicates.

### 2.13. Compressed Juiciness

The compressed juiciness of cooked patties was evaluated following a previous method with slight modifications [5]. Approximately 1 cm^3^ cubes were taken from cooked patties and were placed between two filter papers, followed by pressing for 30 s at 1000 g force using a TA-4 probe (1–1/2-inch diameter acrylic cylinder, 20 mm tall) equipped on a Texture Analyzer (Stable Micro System, Godalming, Surrey, UK). The weight of the samples was recorded before and after the compression and used to calculate the compressed juiciness as follows. Four replicates were tested for each treatment.

(10) $$\mathrm{Compressed}\;\mathrm{juiciness}\;(\%)=\frac{\mathrm{weight}\;\mathrm{of}\;\mathrm{sample}\;\mathrm{before}\;\mathrm{pressing}\;\left(g\right)-\;\mathrm{weight}\;\mathrm{of}\;\mathrm{sample}\;\mathrm{after}\;\mathrm{pressing}\left(g\right)}{\mathrm{weight}\;\mathrm{of}\;\mathrm{sample}\;\mathrm{before}\;\mathrm{pressing}\;\left(g\right)}\;\times \;100$$

### 2.14. Statistical Analysis

Data were analyzed using one-way ANOVA by the SAS University Edition (SAS Institute Inc., Cary, NC, USA). Duncan’s multiple range test was used for mean comparisons, and *p* < 0.05 was considered significantly different. Least significant difference (LSD) values were calculated at 5% level of significance. Pearson correlation coefficients were determined to investigate the relationships among variables.

## 3. Results and Discussion

### 3.1. Proximate Compositions of TVP 

Proximate compositions, including protein, moisture, ash, fat and total carbohydrate contents of TVPs, are presented in Table 1. As shown, TVP samples varied significantly in protein content among the diverse protein sources, with textured pea proteins overall having the highest protein amount (samples 15–21, 62.4 to 76.6%), which was closely followed by textured wheat gluten (samples 22–25, 64.4 to 72.1%) and textured mixed proteins (samples 27–28, 66.3 to 68.3%). Textured soy proteins (samples 1–14, 50.0 to 55.8%) and the textured chickpea protein (50.4%) were the lowest in protein content. Protein is the most paramount component of TVP. A protein content of 50–70% is generally required to form fibrous structures during extrusion [21]. In addition, soy, in comparison with other proteins, such as pea protein, is relatively easier to texturize when forming fibrous structures at lower protein content, as evidenced by the fact that many soy-based TVPs are made from protein concentrates, while pea TVPs are derived from protein isolates [22,23,24]. Overall, the wide range of protein concentration (50.0 to 76.6%) in the studied samples enabled the formation of fibrous textures. 

A similar tendency was also observed in fat content. TVPs derived from pea proteins exhibited a substantially higher fat content (in an average of 6.0%) when compared with textured soy proteins (in an average of 2.7%) or wheat gluten (in an average of 2.8%). The textured mixed proteins located in a high range of fat content (samples 27–28, 6.0 to 6.6%) as well, while the textured chickpea protein (2.0%) was in the lowest range. On the contrary, textured soy proteins registered the highest ash content (5.6 to 7.1%), followed by the descending order of textured pea proteins (3.8 to 5.6%), textured mixed proteins (4.8 to 4.9%), textured chickpea protein (4.6%) and textured wheat gluten (2.4 to 3.0%). The higher ash content possibly arose from a higher amount of minerals in the raw materials prior to texturization. The moisture content of TVPs differed significantly from 4.8 to 8.5%, although with no specific tendency observed among the various protein sources, which might result from the differences in the extrusion conditions and the post-drying processes. The total carbohydrate content was found highly oppositely correlating with protein content (r = −0.984, *p* < 0.01, Table 2. The textured soy proteins (27.4–35.4%) and textured chickpea protein (34.9%) exerted the highest total carbohydrate content. TVPs sourced from wheat gluten (14.7–22.0%) and mixed proteins (14.8–18.2%) contained considerably lower amounts of carbohydrate, whereas textured pea proteins were observed in the lowest place (4.8–20.9%). The variations in chemical compositions of the TVPs are likely responsible for the differences in the physicochemical and textural properties of TVPs before and after formulating to patties.

### 3.2. Physicochemical Characteristics of TVP

Protein solubility commonly functions as a vital indicator of the degree of protein texturization [11]. Upon extrusion cooking, the protein is thermally denatured, with a series of unfolding and aggregation, leading to a decrease in soluble protein. Thus, lower solubility of textured proteins is usually observed compared with their native counterparts [11,20]. The soluble protein content of the studied TVPs ranged significantly between 43.0 and 90.3%, as shown in Table 3. An ascending trend was observed as follows: wheat gluten-based TVPs (samples 22–25, 43.0 to 48.5%) < pea-based TVPs (samples 15–21, 59.7 to 73.5%) < soy-based TVPs (samples 1–14, 74.9 to 90.3%). Meanwhile, the chickpea-based TVP (sample 9, 79.5%) exerted comparable solubility to soy-based TVPs, while the protein solubility of pea/chickpea- (sample 27, 68.6%) and pea/navy bean- (sample 28, 67.1%) mixed protein based TVPs fell within the range of pea-based TVPs. The differences of solubility among the various protein sources may arise out of their intrinsically different molecular structures, as well as varying degrees of protein denaturation during extrusions with diverse conditions. 

A significantly negative relationship existed between the protein solubility and the protein content (r = −0.775, *p* < 0.01), as presented in Table 2. Indeed, a higher protein content could possibly contribute to a greater extent of protein denaturation during extrusion cooking, which resulted in an increase in protein texturization and insoluble proteins, thus lowering the solubility [11,18,20]. Moreover, the intermolecular disulfide bond was suggested as the major force being responsible for the fiber formation of TVP [25,26]. In contrast to legume proteins, wheat gluten contains relatively higher levels of methionine and cysteine [11]. Such sulfur-containing amino acid residues are likely to result in more disulfide cross linkages during texturization, which thereby lead to an increment of molecular weight and the insolubility of proteins [27]. This could possibly explain the lowest protein solubility of the textured wheat gluten samples (in an average of 45.8%) in the current study. However, the structures of extrudates are complex and are usually stabilized by the collective contributions of hydrophobic interactions, hydrogen bonds, disulfide bonds and their interactions [28]. Studies also showed that the importance of non-covalent bonds outweighed covalent bonds [28]. Overall, a lower protein solubility after extrusion is usually concluded as a greater protein denaturation and texturization.

WAC or OAC indicates the ability of a sample to absorb water or oil at the macromolecular level. The amphiphilicity of a protein enables its ability to interact with both water and oil [29]. As such, WAC and OAC are reliant on the availability of polar and non-polar amino acid residues, as well as the protein’s micro- and macro-structures [29]. A lower presence of hydrophilic and polar amino acids over the surface of the protein molecule contributes to lower WAC, while higher availability of hydrophobic residues is responsible for higher OAC. Table 3 shows the WAC of TVPs varying from 1.5 to 2.9 g/g, being independent of protein types or protein contents but potentially associated with the available amounts of polar amino acids in each sample. Meanwhile, an improved entrapment of water has been reported as a consequence of the formation of a protein matrix that is induced by protein denaturation during extrusion [24]. In this study, the wheat-gluten-based TVPs may take great advantages of this phenomenon, as wheat gluten exerted statistically lower protein content but exhibited comparable WAC to that of pea-based TVPs (in an average of 2.1 and 1.9 g/g, respectively). Apart from proteins, the higher carbohydrate contents in the current extrudate samples may also play an important role in the WAC results, since more starch granules were able to absorb more water after gelatinization [9], which might account for the similar WAC of textured soy proteins (in an average of 2.1 g/g) to that of textured pea proteins, although the former were significantly low in protein content (Table 1). 

It is worth noting that OAC was substantially greater for TVPs derived from pea proteins (samples 15–21, 0.82 to 1.04 g/g) than those made with wheat gluten (samples 22–25, 0.75 to 0.86 g/g) or soy proteins (samples 1–14, 0.69 to 0.84), which occurred possibly due to a higher content of hydrophobic amino acids in pea proteins (30.26 g/100 g protein) than in others (28.23 g/100 g protein for wheat gluten and 26.21 g/100 g protein for soy protein), as confirmed by Samard and Ryu [11]. Moreover, OAC was found to positively correlate with fat content (r = 0.852, *p* < 0.01) and protein content (r = 0.711, *p* < 0.01) of TVPs (Table 2). Joshi et al. [30] found that full-fat oilseed flours exhibited lower OAC than their defatted counterparts, as the removal of the fat greatly improved the protein proportion, thus allowing better capillary attraction between the protein and the oil [31]. However, a relatively higher fat content, which was not able to significantly lower the protein content, favored the OAC results in the current study, as the non-polar lipid may enhance the interactions with oil on the basis that protein was the predominant composition governing the OAC of the studied TVPs. On the other hand, WAC and OAC may associate with the extent of denaturation, as extrusion cooking results in the unfolding of proteins and the exposure of more hydrophobic sites [24]. Thus, increasing the protein concentrations may not only contribute to a higher amount of hydrophobic amino acids but is also potentially responsible for the greater extent of protein denaturation induced by extrusion, thereby introducing more available hydrophobic sites, which contribute to greater OAC values. Osen et al. [24] reported that extrusion heat treatment enhanced the OAC of pea protein isolate due to the exposure of more hydrophobic sites. Meanwhile, the polar carbohydrates may, on the other hand, have a negative effect on the extent of interactions with oil, as shown an opposite relationship between carbohydrate content and OAC (r = −0.763, *p* < 0.01, Table 2). In summary, WAC and OAC are multifactor dependent, including protein composition, protein denaturation, as well as the extent of interactions with water and oil [24].

### 3.3. RVA Pasting Properties of TVP

Viscosity plays a crucial role in altering the flow behavior and the mechanical energy input in extrusion cooking [32]. In this study, RVA pasting profiles were obtained to understand the viscosity properties of proteins after texturization. As shown in Table 3, the TVPs behaved dramatically differently upon hydration, heating and cooling under a slow shear. During heating, all samples, regardless of the protein types, endured vast elevation in their viscosities, achieving significantly different peak viscosities ranging from 502 to 4252 cP. However, the peak viscosities were diminished to some extent from the shear in the case of textured wheat gluten (samples 22–25), as indicated by the lower final viscosities compared with their corresponding peak viscosities. Differing from this, the TVPs derived from other sources were increasing in viscosity throughout the holding and cooling, implying their better abilities against shear thinning, while forming viscous pastes or gels upon cooling, which may benefit the texture of the final products. The reduction in the final viscosity could possibly be related to the low protein solubility of textured wheat gluten (Table 3) on the basis of understanding that lower protein solubility is indicative of a more complete texturization, thus a higher denaturation degree, as stated earlier, and the already denatured proteins may have induced weaker protein–protein interactions upon heating, which weakened the resistance to shearing and thereby decreased the final viscosities. 

Despite distinct variations in viscosities, both peak viscosity and final viscosity were found positively correlating with WAC (r = 0.621 and 0.549, respectively, *p* < 0.01, Table 2). This finding is in line with previous studies, where a protein with higher WAC was able to absorb more water, which resulted in higher viscosities [24]. On the other hand, in contrast with pea or wheat gluten, textured soy proteins (samples 1–14) generally required a longer time (ranging from 6.6 to 7.0 min) to achieve peak viscosity, indicating that soy proteins need more time to hydrate and bind water and higher temperatures to denature before reaching the maximum viscosities. This result may be attributed to the relatively higher carbohydrate amount in such samples (Table 1), which may interfere with the hydration and swelling process of proteins, thus retarding the denature time (r = 0.633 between carbohydrate and peak time, *p* < 0.01, Table 2).

### 3.4. Bulk Density 

The bulk density of TVP products interprets the overall expansion and changes in the protein network [12]. The studied TVP samples displayed a wide range in bulk density, as shown in Table 4, with sample 26 (chickpea protein) being the highest (453 g/L), and sample 27 (pea/chickpea mixture proteins) being the lowest (153 g/L). TVPs derived from soy proteins (samples 1–14) generally exhibited higher bulk density, going from 238 to 384 g/L, than those derived from pea proteins (samples 15–21, from 187 to 303 g/L) or wheat gluten (samples 22–25, from 211 to 222 g/L). Conventionally, higher protein content has been shown to undergo a higher degree of protein cross-linking and forming strong structures, which prevents expansion, thus increasing bulk density [33]. However, in this case, the different intrinsic properties of the raw material may make greater contributions to bulk density. As stated earlier, in contrast to other proteins, soy protein usually exerts a better ability to texturize and forms stronger structures, which result in a higher bulk density. Moreover, the wide spectrum of bulk density may also result from other extrusion variables, such as feed moisture, extruder barrel temperature and screw speed [12,13]. 

### 3.5. Rehydration Property 

**Figure 1:**
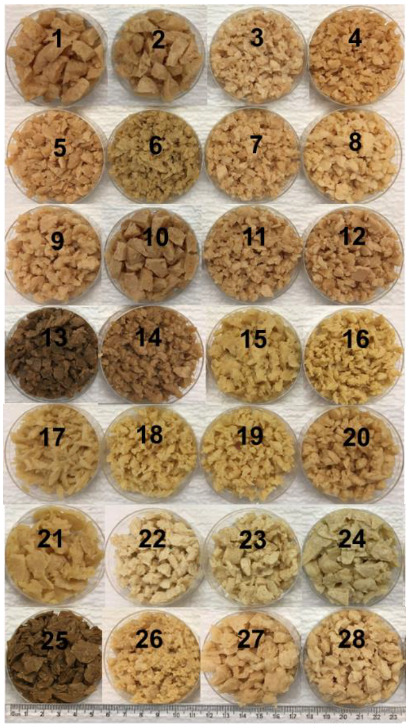
Visible appearance of different commercial TVPs. TVP types: 1–14: textured soy proteins; 15–21: textured pea proteins; 22–25: textured wheat gluten; 26: textured chickpea protein; 27: textured pea/chickpea mixed proteins; 28: textured pea/navy bean mixed proteins.

**Figure 2:**
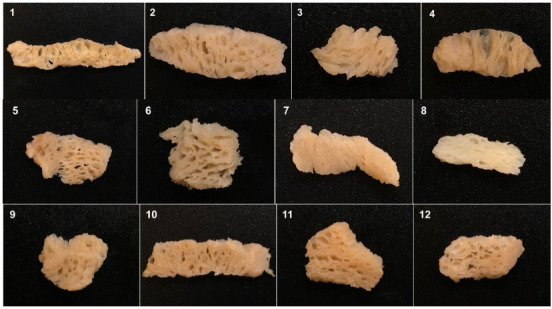

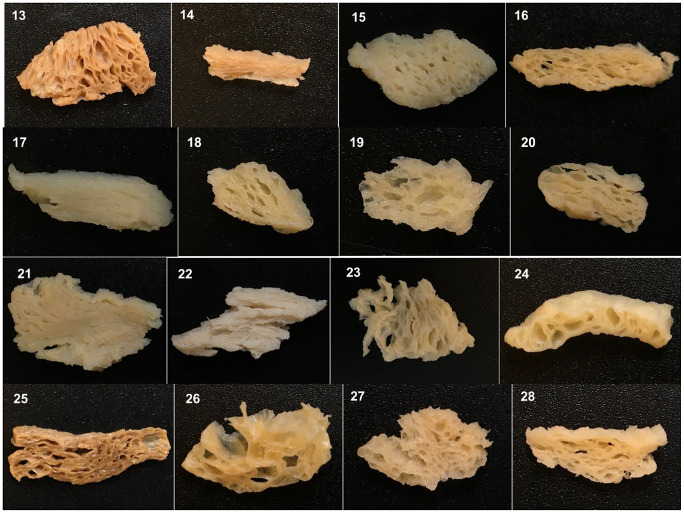
Structures of different commercial TVPs after hydration. TVP types: 1–14: textured soy proteins; 15–21: textured pea proteins; 22–25: textured wheat gluten; 26: textured chickpea protein; 27: textured pea/chickpea mixed proteins; 28: textured pea/navy bean mixed proteins.

Water is critical in meat products to endow the appropriate texture and juiciness, so as to ensure customer acceptability. RHC, referring to the amount of water that could be held by the intact TVP upon rehydration, is an imperative factor affecting the meat-like texture of plant-based meat analogs [9]. In the current study, the RHC values of all samples were significantly different to each other, from 1.5 to 4.2 g/g, as demonstrated in Table 4. Differences in RHC are dependent on protein types, interactions between protein-water molecules, and water–water molecules [11,20] but are more closely related to the product structure, in particular, the porosity and air cell size [9,20]. Here, the external appearance and internal structure of TVPs after hydration are distinguished in Figure 1 and Figure 2. As shown, the TVP samples showed porous structures with various sizes and numbers of air cells, which may have resulted from the different degrees of expansion during extrusion. It is worth mentioning that the images were taken as their naturally displayed directions (longitudinal or horizontal cross sections of extrusion), since the current samples were commercially obtained and were difficult to cut purposely due to the limitation of their shape and size. This could explain why some TVPs exhibited more elongated cells, while others had pores with smaller diameters (Figure 2). Diverging from some previous studies that related a higher RHC to a lower bulk density, as products with low bulk density may possess higher porosity, which allows for faster water uptake and consequently leads to a better water-holding capacity [9,11], no such clear correlation occurred among the current samples. More compact products, such as samples 8, 14, 17, 21, 22, were also able to retain a great amount of water, as evidenced by the relatively high RHC values (3.3 to 3.8 g/g), while lower RHC also occurred in more porous and fibrous structures (samples 1, 2, 6, 10, 13, 16, 24, from 1.5 to 2.5 g/g). The inconsistence may be due to the difference in determining the RHC. The comparatively longer draining time in the current study (1 h) may permit more water to drain off from the more porous protein network, as a higher number of air cells is likely to result in easier water release caused by the gravitational force, whereas a shorter draining time possibly only allows water to drip and evaporate from the surface. In addition to the pore number, the size of the air space is also important to retain water [32].

### 3.6. Textural Properties of TVP

Texture is undoubtedly the most crucial attribute characterizing the quality of textured plant proteins, since a desirable texture that mimics the real meat is the main task of meat analogs. Table 4 shows the textural properties of hydrated TVPs in terms of hardness, resilience, cohesiveness, springiness and chewiness. Hardness is the maximum force required to attain a defined deformation [34]. It differed in a wide range, varying from 400 to 2428 g, among the studied samples (Table 4). Hardness may be indicative of the degree of protein texturization [20]. In this sense, a higher presence of protein content in the starting material is assumed to increase the degree of texturization and protein cross-linking, which prevents further expansion and leads to a higher hardness [20]. Webb et al. found that hardness decreased with the increasing inclusion of chickpea flour, from 10% to 30%, which interfered with the protein–protein interactions [9]. In addition, the hardness and RHC of TVPs appeared to be negatively correlated (r = −0.765, *p* < 0.01, Table 2), agreeing with some previous studies [9,26] that extensive hydration of TVP usually leads to a softer texture [35,36]. Additionally, the diversity of hardness may arise from the various processing variables. Rising barrel temperature and lowering feed moisture have been reported to associate with higher hardness [37]. Overall, it is rather difficult to manifest a clear clue addressing the wide range of hardness here, since all the studied samples came from different commercial sources and were made under diverse extrusion conditions.

Resilience measures how a sample recovers from deformation with regard to speed and forces. As shown in Table 4, resilience values extended from 16.4 to 37.5%, displaying no specific tendency among the protein sources, although being inversely correlated with bulk density (r = −0.665, *p* < 0.01, Table 2). Products with higher bulk density potentially possess more compact structures, which likely impair the resilience. Here, TVP samples exhibited relatively high springiness, going from 79.1 to 100.5%, suggesting good abilities of TVPs to regain their original form after compression. Likewise, springiness was negatively related to bulk density, with r = −0.724 (*p* < 0.01, Table 2). The lower bulk density benefits a higher porosity and loose structure, thereby enhancing the springiness. Cohesiveness indicates the strength of internal bonds and inter- and intra-actions constituting the product [34]. Samples exhibited a cohesiveness of 0.53 to 0.72 in the current study, which might be a response to the different degree of interactions formed during texturization and rehydration [12]. In addition, chewiness represents the energy necessary to masticate a solid product for swallowing [34]. As expected, the wide spectrum of chewiness (276 to 1530 g) positively corresponded with hardness (r = 0.977, *p* < 0.01). The lower chewiness may largely be a result of a higher RHC, which leads to a softer texture (r = −0.737 between chewiness and RHC, *p* < 0.01, Table 2). 

### 3.7. Cooking Properties of TVP-Based Patties

**Figure 3:**
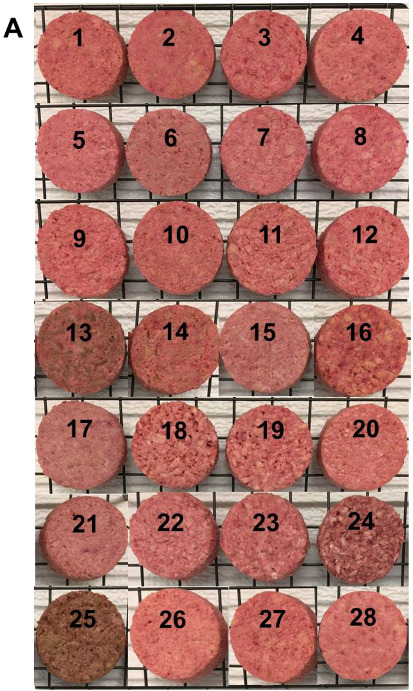

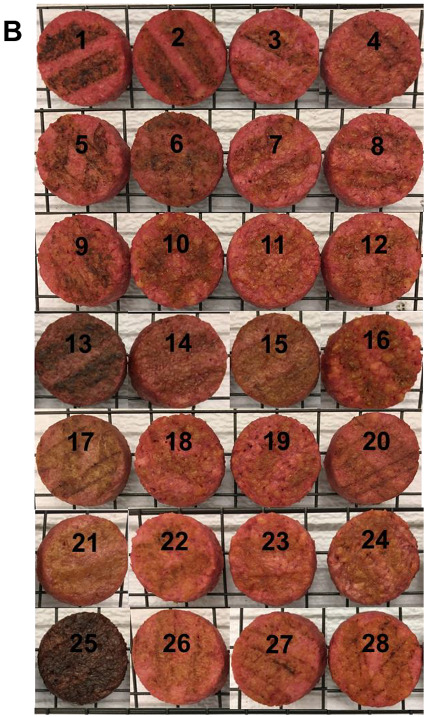
Pictures of TVP-based patties before (**A**) and after (**B**) cooking. Patty types: 1–14 produced from textured soy proteins; 15–21 from textured pea proteins; 22–25 from textured wheat gluten; 26 from textured chickpea protein; 27 made from textured pea/chickpea mixed proteins; and 28 made from textured pea/navy bean mixed proteins.

The visible appearance of TVP-based patties before and after cooking is presented in Figure 3. It is worth mentioning that the patties in this study had the same formulation. In addition to the different types of TVPs, all the other ingredients (salt, pigment, binder, etc.) were added in the same amounts. Thus, the diverse properties of patties were assumed to result from the various properties of the TVPs. The effect of cooking on patties was investigated by measuring cooking loss, diameter shrinkage, moisture retention and fat retention. Cooking loss is an important parameter evaluating the textural and sensorial attributes of meat products with regard to juiciness, tenderness and also the yield of the final product [35]. It is mainly caused by the loss of liquid (moisture and fat) during the cooking process [38] and is linked to different variables, such as cooking time, temperature and method, type and amount of particular ingredients in the formulation [39,40]. 

The cooking loss of TVP-based patties ranged vastly from 11.6 to 18.5% (Table 5), irrespective of protein types. A positive relationship was observed between the RHC of TVPs and the cooking loss, as stated in Table 2 (r = 0.679, *p* < 0.01). At higher RHC, a relatively higher amount of water was introduced to the meatless patty, causing the proportional decrease in solid content on the basis that the same total amount of hydrated TVP was incorporated. Upon heating, the hydrophobic residues in the proteins became exposed; the heated TVP consequently contributed less hydrophilic interactions with water, which resulted in a leakage of water, and thus, a high cooking loss [35]. On the other hand, the methylcellulose in the formulation served as a binder that created a network upon protein hydration and helped combining the ingredients together [7]. It is supposed that the cage-like water molecules encircle the hydrophobic methyl residues of the methylcellulose polymer. Nevertheless, the increasing temperature disrupts the cage structure, causing the release of water [41]. In light of this, a higher cooking loss is likely to occur in patties formed by TVPs with higher RHC. This finding is in accordance with many other studies. Wi et al. [35] found a typical increase in cooking loss from 12.5 to 14.5% as the amount of water increased in meat analogs. The same trend was also reported by Sakai et al. [41], where the increasing amount of added water elevated the cooking loss. 

It is also interesting to note that patty cooking loss was positively associated with protein viscosities (r = 0.605 and 0.660 for peak and final viscosity, respectively, *p* < 0.01, Table 2). In this case, it might be hypothesized that the enhanced hydrophobic interactions induced by protein denaturation upon heating helped form a tighter network, which not only increased the viscosity but also decreased the free space within the protein matrix, thus reducing water penetration and uptake and increasing the cooking loss.

Cooking causes meat shrinkage due to protein denaturation, change of structure, moisture loss and fat drainage [42]. As expected, the reduction in patty diameter was highly correlated with cooking loss (r = 0.786, *p* < 0.01, Table 2), together with a positive correlation with RHC (r = 0.679, *p* < 0.01, Table 2), which ranged from 4.4 to 9.5% (Table 5). This degree of shrinkage fell within the spectrum of 3.6–12.3% for commercial textured vegetable protein (C-TVP) and textured isolate soy protein (T-ISP) based patties, as reported by Bakhsh and others [43].

Proteins form a gel matrix during the cooking treatment, which is able to retain the essential components [42]. Moisture and fat retentions refer to the capabilities of a product to retain water and fat after cooking. They are crucial factors ensuring the sensory quality and acceptability of meat products. Table 5 displays the moisture retention of TVP-based patties varying from 73.2 to 80.5%, while the fat retention differs from 74.3 to 92.4%, being unaffected by protein sources. The diversity of these parameters was possibly derived from the different degrees of protein denaturation and the extent of the interactions between water/oil and the TVP structure [18]. Both moisture retention and fat retention were inversely related to cooking loss (r = −0.655 and r = −0.684, respectively, *p* < 0.01, Table 2), as a higher cooking loss usually occurs when a patty loses more fat or moisture [38,42]. In addition, the negative correlation between moisture retention and protein viscosity (r = −0.530, *p* < 0.01, Table 2) may again give an insight into the enhancement of the hydrophobic bindings, which allowed rising viscosity and retaining less moisture. Meanwhile, less fat was likely to be held due to less free space and enhanced rigidity of the protein gel, which may help explain the negative relationship between fat retention and pasting viscosity (r = −0.601 and r = −0.552 for peak and final viscosity, respectively, *p* < 0.01, Table 2). However, fat retention is a complex parameter, which may be associated with several other chemical and physical mechanisms [38]. 

### 3.8. Textural Properties of TVP-Based Patties

Table 6 shows the textural properties of cooked patties derived from different TVPs. While hardness in the patty form was highly related to that in the hydrated counterparts (r = 0.885, *p* < 0.01, Table 2), the former was generally greater than the latter (559 to 2767 g vs. 400 to 2427 g), which was possibly due to the methylcellulose binding during the patty formation and gelling during cooking that resulted in the compacting of the material. During the cooking process, methylcellulose gradually loses its hydrated water and is likely to bind together owing to the extensive hydrophobic interactions, which highly favors the thermal formation of gels [43]. The strong gels thereby toughen the texture of the final product. Consistent with TVP hardness, the hardness in cooked patties varied negatively with the RHC of TVPs (r = −0.791, *p* < 0.01, Table 2), since a higher water content commonly forms more softened meat analogs [35,36]. Meanwhile, when TVPs with higher RHC were incorporated, a relatively lower solid content was induced to the patty. The decrease in the solid amount may have caused the reduction in hardness as well. However, disagreeing with some previous studies [44], the hardness in the current patties was inversely associated with cooking loss (r = −0.618, *p* < 0.01, Table 2). It is possible that TVPs with a high RHC, although undergoing a higher cooking loss, as previously stated, may have still retained a relatively higher amount of water, and the softening effect caused by the residual water played a more important role than the toughening impact induced by the shrinkage, which thereby resulted in a lower hardness in such samples compared with those with a lower RHC but also lower cooking loss. 

A moderately negative correlation existed between patty hardness and peak viscosity (r = −0.599, *p* < 0.01, Table 2). Given the above explanation, the comparatively higher water remainder in samples with high RHC may not only result in a tender texture of a patty, but also contribute to a relatively higher viscosity of the protein due to a higher retention of water during cooking. It is also worth noting that higher hardness was related to an increase in fat retention (r = 0.537, *p* < 0.01, Table 2). Barbut and Marangoni reported that oil droplets could help connect the protein–protein interactions due to their smaller size but larger surface area [45]. Therefore, an increasing oil globule in products with higher fat retention incremented such hydrophobic linkage and formed a more compact and firmer gel network among the protein matrix, thus enhancing the resistance to compression.

Differing from hardness, other textural attributes were all found to reduce in the patty form in contrast with the hydrated TVPs before binding (Table 4 and Table 6). The resilience of the cooked patties remained with substantially lower values, going from 4.1 to 11.5% (Table 6). The observed lower results in patties made from textured soy proteins could be attributed to the better ability of soy protein to form strong structures, thus a more compact texture and higher bulk density (r = −0.506 between resilience and bulk density, *p* < 0.01, Table 2). Similarly, a dramatic decrease in springiness was observed, as most values ranged from 50 to 80% (Table 6), implying that they were more prone to be deformed in the patty form. Apart from a more compacted form induced by methylcellulose binding, the fat content introduced in the formulation that helped fill the interspace within the protein matrix may have also resulted in a reduction in springiness. Cohesiveness was similar to the above, in that there was a decline from the hydrated extrudates to the patty form (0.53–0.72 vs. 0.19–0.38). Cohesiveness is related to intermolecular attractions, which are able to hold the elements together [44]. In a food product, cohesiveness also represents the extent to which the food can be deformed before it ruptures [44]. Here, it may be more useful to regard the cohesiveness of the cooked patties as the strength to withstand fracture in a patty as an entirety rather than to disintegrate the TVP particles, which resulted in the difference before and after the formation of patties. As for chewiness, significantly lower values were found in the patty form (93 to 391 g) compared with those of hydrated TVPs before binding (208 to 1530 g). The lower force required to chew the cooked patties was possibly due to the protein denaturation caused by the cooking treatment, which altered the protein conformation and structure.

Shear force represents the maximal force needed to cut a patty, which can be interpreted as an indirect measurement of product tenderness [5]. Here, the shear forces ranged from 69 to 527 g among the studied patties. As reported elsewhere [43], shear force behaved in a significantly similar manner to hardness (r = 0.778, *p* < 0.01, Table 2), with RHC being the predominant affecting factor in the current study (r = −0.621, *p* < 0.01, Table 2). In this respect, a higher RHC of the TVP would be a favorable implication, achieving lower hardness and shear force, thus a softer and more tender texture.

Compressed juiciness refers to the percentage weight loss of cooked patties established in a compression test. As found in Table 6, juiciness in cooked patties varied significantly, from 4.0 to 10.3%. In general, TVPs with higher RHC yielded more juices when formulating a patty, as evidenced by a significantly positive correlation between the RHC and juiciness (r = 0.812, *p* < 0.01, Table 2). This phenomenon was inevitable due to a relatively higher amount of water left within the protein matrix, which was able to be squeezed out. Moreover, the compressed juiciness was negatively correlated to hardness, chewiness and shear force of patties (r = −0.883, −0.540 and −0.653, respectively, *p* < 0.01, Table 2), since a firmer structure was more capable of retaining fluid and more resistant to compression, thus imparting less juice [46]. Overall, the physicochemical and functional properties of the proteins, the ingredients in patty formulation, as well as the cooking process all play important roles in carrying over the TVP properties into the textures of final products.

## 4. Conclusions

Twenty-eight commercial textured vegetable proteins derived from different protein types and sources were comprehensively analyzed with respect to proximate compositions, physicochemical and functional properties of raw TVPs, alongside the cooking and textural characteristics of the final meatless patties. Significant correlations were established between the upstream and downstream attributes. Variations in chemical compositions were the basis contributing to different physicochemical and functional properties of TVPs. Protein content was found to be important in determining protein solubility (r = −0.775, *p* < 0.01), while fat content was crucial to OAC (r = 0.852, *p* < 0.01). Meanwhile, the WAC of the TVP powder played an important role in the pasting property (r = 0.621, and 0.549 for peak viscosity and final viscosity, respectively, *p* < 0.01). The bulk density of TVPs in this study was primarily determined by the intrinsic property of the material types rather than other parameters. The diversity in the functional properties of TVPs resulted in various textures. As was found, higher RHC imparted lower hardness and chewiness of hydrated TVPs (r = −0.765 and −0.737, respectively, *p* < 0.01), while TVPs with lower bulk density exhibited higher resilience and springiness (r = −0.665 and −0.724, respectively, *p* < 0.01). The versatile attributes of raw TVPs were further carried over into the final patties. The cooking loss and textural properties (hardness, shear force and compressed juiciness) of meatless patties were predominantly associated with RHC (r = 0.679, −0.791, −0.621 and 0.812, respectively, *p* < 0.01). Aside from that, the pasting property of TVPs also served as an important indicator of patty attributes, as significant correlations occurred accordingly. Moreover, binders such as methylcellulose played important roles in integrating TVPs into the final products, causing significant differences in textures before and after binding. As such, targeting the texture of the final products depends not exclusively on the raw TVP but also on the binding system. The present study, for the first time, provided a systematic evaluation correlating the physicochemical and functional properties of raw TVPs to the cooking and textural properties of the final meatless patties. These findings may help provide a bottom-up insight for designing TVPs with various characteristics, which may benefit the final desirable meat analogs. Further studies, such as using other types of meat analogs and conducting consumer sensory evaluations, are suggested to further validate the importance of the correlations discovered in this study and unveil the possible associations between raw TVPs and sensory attributes, thus helping to improve consumer acceptability.

## Figures and Tables

**Table 1:** Proximate composition (as-is wet basis) of different TVPs.

Sample ^A^	Protein Content (%)	Moisture Content (%)	Ash Content (%)	Fat Content (%)	Total Carbohydrate (%)
1	51.1 ± 0.1 ^no^	7.2 ± 0.08 ^f^	6.1 ± 0.02 ^g^	2.2 ± 0.3 ^jkl^	33.4
2	51.4 ± 0.2 ^mn^	8.1 ± 0.03 ^bc^	6.0 ± 0.03 ^h^	2.5 ± 0.1 ^hi^	32.0
3	51.1 ± 0.0 ^no^	7.1 ± 0.09 ^g^	6.2 ± 0.01 ^f^	2.3 ± 0.0 ^ijk^	33.4
4	52.1 ± 0.1 ^l^	6.5 ± 0.07 ^k^	6.3 ± 0.01 ^d^	2.2 ± 0.0 ^klm^	32.9
5	51.3 ± 0.2 ^mn^	6.1 ± 0.06 ^m^	6.5 ± 0.04 ^b^	2.2 ± 0.2 ^ijk^	33.9
6	55.7 ± 0.1 ^k^	7.3 ± 0.07 ^e^	7.1 ± 0.01 ^a^	2.4 ± 0.1 ^hij^	27.4
7	50.5 ± 0.5 ^pq^	7.8 ± 0.02 ^d^	6.4 ± 0.00 ^cd^	2.1 ± 0.1 ^klm^	33.2
8	51.0 ± 0.0 ^no^	7.2 ± 0.02 ^f^	5.6 ± 0.04 ^i^	7.9 ± 0.2 ^b^	28.3
9	50.0 ± 0.0 ^q^	6.9 ± 0.03 ^h^	6.4 ± 0.02 ^c^	2.2 ± 0.0 ^ijk^	34.4
10	51.5 ± 0.0 ^mn^	7.8 ± 0.01 ^d^	6.3 ± 0.02 ^e^	1.9 ± 0.2 ^m^	32.5
11	51.7 ± 0.1 ^lm^	8.1 ± 0.02 ^bc^	6.3 ± 0.02 ^e^	2.3 ± 0.0 ^ijk^	31.6
12	50.5 ± 0.1 ^p^	6.7 ± 0.08 ^i^	6.5 ± 0.02 ^b^	2.1 ± 0.0 ^klm^	34.2
13	50.7 ± 0.0 ^op^	6.6 ± 0.01 ^j^	6.2 ± 0.06 ^f^	2.4 ± 0.1 ^hij^	34.1
14	50.5 ± 0.0 ^p^	5.4 ± 0.02 ^p^	6.2 ± 0.02 ^f^	2.5 ± 0.0 ^h^	35.4
15	76.6 ± 0.3 ^a^	5.8 ± 0.01 ^o^	3.9 ± 0.02 ^o^	4.9 ± 0.2 ^e^	8.8
16	74.0 ± 0.3 ^d^	6.0 ± 0.02 ^n^	5.6 ± 0.01 ^i^	5.0 ± 0.1 ^e^	9.4
17	74.1 ± 0.2 ^cd^	7.3 ± 0.03 ^e^	5.5 ± 0.03 ^j^	4.8 ± 0.2 ^e^	8.3
18	75.1 ± 0.4 ^b^	6.2 ± 0.01 ^l^	5.4 ± 0.01 ^j^	8.5 ± 0.1 ^a^	4.8
19	74.5 ± 0.1 ^c^	7.0 ± 0.02 ^g^	5.1 ± 0.00 ^k^	8.1 ± 0.0 ^b^	5.3
20	62.4 ± 0.2 ^j^	6.9 ± 0.03 ^h^	3.8 ± 0.03 ^p^	6.0 ± 0.2 ^d^	20.9
21	71.8 ± 0.1 ^e^	8.1 ± 0.04 ^bc^	4.9 ± 0.04 ^l^	4.9 ± 0.2 ^e^	10.2
22	66.1 ± 0.4 ^h^	8.2 ± 0.04 ^b^	2.4 ± 0.01 ^t^	2.8 ± 0.1 ^g^	20.5
23	70.5 ± 0.1 ^f^	8.1 ± 0.03 ^c^	2.6 ± 0.08 ^s^	3.1 ± 0.1 ^f^	15.7
24	72.1 ± 0.1 ^e^	7.3 ± 0.03 ^e^	3.0 ± 0.04 ^q^	2.9 ± 0.1 ^fg^	14.7
25	64.4 ± 0.3 ^i^	8.5 ± 0.05 ^a^	2.7 ± 0.03 ^r^	2.4 ± 0.0 ^hij^	22.0
26	50.4 ± 0.0 ^pq^	8.1 ± 0.01 ^bc^	4.6 ± 0.01 ^n^	2.0 ± 0.2 ^lm^	34.9
27	68.3 ± 0.1 ^g^	5.5 ± 0.03 ^p^	4.9 ± 0.03 ^l^	6.6 ± 0.1 ^c^	14.8
28	66.2 ± 0.2 ^h^	4.8 ± 0.01 ^q^	4.8 ± 0.04 ^m^	6.0 ± 0.1 ^d^	18.2
Ave. soy	51.4 ± 1.4 c	7.9 ± 0.8 b	6.3 ± 0.3 a	2.7 ± 1.5 b	32.6 ± 2.3 a
Ave. pea	72.7 ± 4.7 a	6.8 ± 0.8 b	4.9 ± 0.8 b	6.0 ± 1.6 a	9.7 ± 5.4 c
Ave. wheat	68.3 ± 3.6 b	8.0 ± 0.5 a	2.7 ± 0.2 c	2.8 ± 0.3 b	18.2 ± 3.6 b
Average ^B^	60.2	7.0	5.3	3.8	23.8
LSD (5%) ^C^	0.4	0.1	0.1	0.2	-

^A^ Protein types of samples: 1–14 (soy protein), 15–21 (pea protein), 22–25 (wheat gluten), 26 (chickpea protein), 27 (pea and chickpea protein mixture), 28 (pea and navy bean protein mixture). Means with different superscript letters within the same column are significantly different (*p* < 0.05) among samples 1–28. Different lowercase letters indicate significant difference among means of soy, pea and wheat gluten samples within the same column (*p* < 0.05). ^B,C^ Average values of all samples and least significant difference (LSD) for comparison of different samples.

**Table 2:** Pearson correlation coefficients ^®^ for the relationships between properties of TVPs and TVP-based patties.

	PC	MC	AC	FC	CC	BD	RHC	WAC	OAC	PS	PV	PT	FV	T-H	T-R	T-CO	T-S	T-CH
PC	1	−0.162	−0.611**	0.628 **	−0.984 **	−0.661 **	0.237	−0.073	0.711 **	−0.775 **	0.057	−0.652 **	−0.274	−0.237	0.386 *	0.057	0.431 *	−0.182
MC		1	−0.221	−0.380 *	0.164	0.094	−0.226	0.250	−0.394 *	−0.191	0.055	−0.294	−0.190	0.095	−0.016	−0.163	0.035	0.080
AC			1	−0.205	0.518 **	0.529 **	−0.160	0.180	−0.351	0.857 **	−0.096	0.620 **	0.416 *	0.340	−0.270	0.014	−0.403 *	0.305
FC				1	−0.732 **	−0.609 **	0.374 *	−0.383 *	0.852 **	−0.195	−0.201	−0.321	−0.259	−0.170	0.214	0.418 *	0.342	−0.102
CC					1	0.674 **	−0.258	0.099	−0.763 **	0.687 **	−0.008	0.633 **	0.277	0.208	−0.375 *	−0.122	−0.431 *	0.149
BD						1	−0.221	0.393 *	−0.552 **	0.591 **	0.225	0.300	0.509 **	0.141	−0.665 **	−0.607 **	−0.724 **	−0.002
RHC							1	0.032	0.362	−0.019	0.554 **	−0.190	0.404 *	−0.765 **	−0.149	0.062	0.107	−0.737 **
WAC								1	−0.337	0.023	0.621 **	−0.041	0.549 **	0.102	−0.113	−0.446 *	−0.082	0.049
OAC									1	−0.325	−0.071	−0.513 **	−0.249	−0.234	0.239	0.312	0.181	−0.188
PS										1	−0.133	0.615 **	0.388 *	0.203	−0.387 *	0.084	−0.414 *	0.170
PV											1	−0.199	0.778 **	−0.470 *	−0.190	−0.470 *	−0.131	0.537 **
PT												1	0.190	0.271	−0.091	0.166	−0.127	0.273
FV													1	−0.277	−0.368	−0.432 *	−0.336	−0.361
T-H														1	0.217	0.192	0.025	0.977 **
T-R															1	0.646 **	0.786 **	0.343
T-CO																1	0.634 **	0.335
T-S																	1	0.167
T-CH																		1
	**CL**		**DS**	**MR**		**FR**	**CJ**		**P-H**	**P-R**	**P-CO**		**P-S**		**P-CH**		**SF**	
PC	−0.073		−0.047	0.046		−0.173	0.149		−0.102	0.577 **	0.519 **		0.515 **		0.434 *		−0.094	
MC	−0.302		−0.305	0.222		0.148	−0.033		0.050	0.078	0.411 *		0.372		0.249		0.097	
AC	0.111		−0.016	−0.357		0.189	−0.257		0.318	−0.382 *	−0.620 **		−0.636 **		−0.252		0.273	
FC	0.104		0.099	−0.116		−0.111	0.153		−0.108	0.279	0.214		0.216		0.181		0.009	
CC	0.063		0.056	0.003		0.150	−0.136		0.074	−0.564 **	−0.497 **		−0.488 **		−0.440 **		0.045	
BD	0.076		0.032	−0.105		0.040	−0.105		0.023	−0.506 **	−0.514 **		−0.323		−0.444 *		−0.147	
RHC	0.679 **		0.648 **	−0.290		−0.415 *	0.812 **		−0.791 **	−0.147	0.405 *		0.387 *		−0.412 *		−0.621 **	
WAC	0.341		0.076	−0.508 **		−0.361	0.177		−0.068	−0.196	−0.023		0.018		−0.242		−0.204	
OAC	−0.007		−0.005	−0.088		−0.071	0.071		−0.104	0.419 *	0.191		0.286		0.255		−0.112	
PS	0.243		0.137	−0.319		0.123	−0.147		0.108	−0.604 **	−0.686 **		−0.624 **		−0.488 **		0.066	
PV	0.605 **		0.478 *	−0.391 *		−0.601 **	0.579 **		−0.599 **	−0.168	0.295		0.220		−0.396 *		−0.587 **	
PT	0.116		0.134	−0.143		0.065	−0.191		0.186	−0.414 *	−0.528 **		−0.663 **		−0.360		0.189	
FV	0.660 **		0.456 *	−0.530 **		−0.552 **	0.368		−0.443 *	−0.497 **	−0.185		−0.200		−0.629 **		−0.479 **	
T-H	−0.495 **		−0.622 **	0.051		0.347	−0.722 **		0.885 **	0.076	−0.458 *		−0.536 **		0.365		0.639 **	
T-R	−0.074		−0.309	−0.155		0.028	−0.408 *		0.398 *	0.595 **	0.244		−0.013		0.606 **		0.321	
T-CO	−0.035		−0.210	−0.015		0.252	−0.212		0.261	0.246	0.027		−0.148		0.299		0.293	
T-S	0.126		−0.090	−0.056		−0.082	−0.006		0.079	0.416 *	0.446 *		0.218		0.385 *		0.089	
T-CH	−0.497 **		−0.644 **	0.077		0.401 *	−0.719 **		0.889 **	0.128	−0.416 *		−0.520 **		0.410 *		0.673 **	
CL	1		0.786 **	−0.655 **		−0.684 **	0.595 **		−0.618 **	−0.445 *	0.012		−0.054		−0.634 **		−0.528 **	
DS			1	−0.333		−0.528 **	0.721 **		−0.714 **	−0.412 *	0.047		−0.050		−0.654 **		−0.489 **	
MR				1		0.560 **	−0.074		0.118	0.257	0.199		0.230		0.327		0.157	
FR						1	−0.437 *		0.537 **	0.390 *	−0.179		−0.079		0.466 *		0.405 *	
CJ							1		−0.883 **	−0.312	0.470 *		0.454 *		−0.540 **		−0.653 **	
P-H									1	0.387 *	−0.379 *		−0.443 *		0.639 **		0.778 **	
P-R										1	0.477 *		0.404 *		0.888 **		0.246	
P-CO											1		0.819 **		0.388 *		−0.215	
P-S													1		0.280		−0.400 *	
P-CH															1		0.543 **	
SF																	1	

Abbreviations: PC, Protein content; MC, Moisture content; AC, Ash content; FC, Fat content; BD, Bulk density; RHC, Rehydration capacity; WAC, Water absorption capacity; OAC, Oil absorption capacity; PS, Protein solubility; PV, Peak viscosity; FV, Final viscosity; T-H, TVP hardness; T-R, TVP resilience; T-CO, TVP cohesiveness; T-S, TVP springiness; T-CH, TVP chewiness; CL, Cooking loss; DS, Diameter shrinkage; MR, Moisture retention; FR, Fat retention; CJ, Compressed juiciness; P-H, Patty hardness; P-R, Patty resilience; P-CO, Patty cohesiveness; P-S, Patty springiness; P-CH, Patty chewiness; SF, Shear force. * *p* < 0.05; ** *p* < 0.01.

**Table 3:** Physicochemical properties of TVPs.

Sample ^A^	WAC (g/g)	OAC (g/g)	Solubility (%)	Pasting Property		
				Peak Viscosity (cP)	Peak Time (min)	Final Viscosity (cP)
1	2.1 ± 0.01 ^ij^	0.69 ± 0.01 ^t^	81.4 ± 0.2 ^e^	544 ± 1 ^q^	7.0 ± 0.0 ^a^	1263 ± 17 ^o^
2	2.1 ± 0.00 ^fgh^	0.71 ± 0.01 ^rs^	77.1 ± 0.6 ^j^	651 ± 4 ^p^	7.0 ± 0.0 ^a^	1519 ± 4 ^n^
3	2.2 ± 0.02 ^efg^	0.77 ± 0.00 ^l^	77.6 ± 0.1 ^j^	2786 ± 4 ^g^	7.0 ± 0.0 ^a^	5832 ± 52 ^d^
4	2.1 ± 0.05 ^gh^	0.70 ± 0.01 ^rs^	82.9 ± 0.1 ^d^	2848 ± 9 ^g^	7.0 ± 0.0 ^a^	6049 ± 8 ^c^
5	2.2 ± 0.03 ^ef^	0.74 ± 0.02 ^nop^	83.6 ± 0.1 ^c^	2938 ± 9 ^f^	7.0 ± 0.0 ^a^	6674 ± 60 ^b^
6	2.3 ± 0.01 ^d^	0.78 ± 0.01 ^k^	81.5 ± 0.2 ^e^	1448 ± 16 ^m^	7.0 ± 0.0 ^a^	2415 ± 35 ^k^
7	2.1 ± 0.02 ^hi^	0.74 ± 0.01 ^op^	80.5 ± 0.2 ^f^	3168 ± 10 ^e^	7.0 ± 0.0 ^a^	6088 ± 16 ^c^
8	2.0 ± 0.02 ^l^	0.84 ± 0.00 ^g^	85.0 ± 0.1 ^b^	1703 ± 18 ^k^	6.6 ± 0.1 ^a^	2876 ± 31 ^j^
9	1.9 ± 0.02 ^l^	0.76 ± 0.00 ^lm^	90.3 ± 0.1 ^a^	2084 ± 16 ^i^	7.0 ± 0.0 ^a^	4615 ± 24 ^g^
10	2.2 ± 0.00 ^e^	0.69 ± 0.01 ^st^	74.9 ± 0.3 ^k^	502 ± 4 ^q^	7.0 ± 0.0 ^a^	1305 ± 3 ^o^
11	2.2 ± 0.04 ^fg^	0.76 ± 0.01 ^lmn^	85.3 ± 0.1 ^b^	2132 ± 17 ^i^	7.0 ± 0.0 ^a^	4962 ± 18 ^f^
12	2.1 ± 0.02 ^jk^	0.73 ± 0.01 ^pq^	85.1 ± 0.1 ^b^	2678 ± 29 ^h^	7.0 ± 0.0 ^a^	6901 ± 30 ^a^
13	1.9 ± 0.02 ^l^	0.72 ± 0.01 ^qr^	78.2 ± 0.1 ^i^	2152 ± 16 ^i^	7.0 ± 0.0 ^a^	4470 ± 39 ^h^
14	2.0 ± 0.02 ^k^	0.80 ± 0.00 ^jk^	78.8 ± 0.3 ^h^	2682 ± 21 ^h^	7.0 ± 0.0 ^a^	5240 ± 53 ^e^
15	2.9 ± 0.05 ^a^	0.92 ± 0.00 ^d^	62.8 ± 0.3 ^p^	4175 ± 159 ^b^	4.8 ± 0.6 ^de^	6957 ± 56 ^a^
16	2.0 ± 0.01 ^jk^	0.95 ± 0.01 ^c^	60.6 ± 0.1 ^q^	546 ± 1 ^q^	5.0 ± 0.1 ^bcd^	596 ± 3 ^r^
17	2.6 ± 0.02 ^b^	0.83 ± 0.01 ^gh^	60.8 ± 0.2 ^q^	3767 ± 1 ^c^	4.5 ± 0.3 ^ef^	4958 ± 59 ^f^
18	1.5 ± 0.02 ^q^	0.98 ± 0.01 ^b^	68.0 ± 0.2 ^n^	532 ± 1 ^q^	7.0 ± 0.0 ^a^	1024 ± 3 ^p^
19	1.5 ± 0.01 ^p^	1.04 ± 0.01 ^a^	68.0 ± 0.0 ^n^	1792 ± 4 ^j^	2.2 ± 0.5 ^g^	2969 ± 8 ^i^
20	1.7 ± 0.01 ^o^	0.97 ± 0.01 ^b^	73.5 ± 0.0 ^l^	659 ± 7 ^p^	5.3 ± 0.0 ^b^	848 ± 1 ^q^
21	2.5 ± 0.04 ^c^	0.82 ± 0.01 ^hi^	59.7 ± 0.4 ^r^	3488 ± 31 ^d^	4.3 ± 0.0 ^f^	5822 ± 30 ^d^
22	2.1 ± 0.01 ^ij^	0.86 ± 0.01 ^f^	47.0 ± 0.2 ^t^	4252 ± 52 ^a^	4.7 ± 0.0 ^de^	2424 ± 20 ^k^
23	1.7 ± 0.02 ^n^	0.76 ± 0.01 ^lm^	48.5 ± 0.0 ^s^	1061 ± 11 ^o^	4.9 ± 0.0 ^cde^	867 ± 4 ^q^
24	1.7 ± 0.03 ^o^	0.75 ± 0.01 ^mno^	44.7 ± 0.6 ^u^	1353 ± 8 ^n^	5.2 ± 0.0 ^bc^	1061 ± 10 ^p^
25	2.2 ± 0.01 ^e^	0.80 ± 0.01 ^ij^	43.0 ± 0.4 ^v^	2713 ± 1 ^h^	4.8 ± 0.1 ^cde^	1829 ± 8 ^l^
26	1.8 ± 0.01 ^m^	0.79 ± 0.00 ^jk^	79.5 ± 0.1 ^g^	1348 ± 6 ^n^	4.2 ± 0.1 ^f^	1581 ± 12 ^m^
27	1.6 ± 0.02 ^p^	0.90 ± 0.00 ^e^	68.6 ± 0.2 ^m^	1120 ± 0 ^o^	7.0 ± 0.0 ^a^	1783 ± 4 ^l^
28	1.5 ± 0.02 ^p^	0.92 ± 0.01 ^de^	67.1 ± 0.3 ^o^	1541 ± 13 ^l^	7.0 ± 0.0 ^a^	2913 ± 1 ^ij^
Ave. soy	2.1 ± 0.1 a	0.74 ± 0.04 b	81.6 ± 4.1 a	2022 ± 925 a	7.0 ± 0.1 a	4300 ± 2037 a
Ave. pea	2.1 ± 0.6 a	0.93 ± 0.08 a	64.8 ± 5.2 b	2137 ± 1635 a	4.7 ± 1.4 b	3310 ± 2617 ab
Ave. wheat	1.9 ± 0.3 a	0.79 ± 0.05 b	45.8 ± 2.5 c	2345 ± 1461 a	4.9 ± 0.2 b	1545 ± 718 b
Average ^B^	2.0	0.81	71.2	2024	6.0	3423
LSD (5%) ^C^	0.04	0.02	0.5	70	0.4	59

Abbreviations: WAC, water absorption capacity of TVP powder; OAC, oil absorption capacity of TVP powder. ^A^ Protein types of samples: 1–14 (soy protein), 15–21 (pea protein), 22–25 (wheat gluten), 26 (chickpea protein), 27 (pea and chickpea protein mixture), 28 (pea and navy bean protein mixture). Means with different superscript letters within the same column are significantly different (*p* < 0.05) among samples 1–28. Different lowercase letters indicate significant difference among means of soy, pea and wheat gluten samples within the same column (*p* < 0.05). ^B,C^ Average values of all samples and least significant difference (LSD) for comparison of different samples.

**Table 4:** Rehydration and textural properties of TVP.

Sample ^A^	Bulk Density (g/L)	RHC (g/g)	Textural Property				
			Hardness (g)	Resilience (%)	Cohesiveness	Springiness (%)	Chewiness (g)
1	238 ± 6 ^kl^	2.4 ± 0.03 ^ij^	1061 ± 79 ^d^	36.2 ± 0.5 ^ab^	0.72 ± 0.02 ^a^	100.1 ± 4.6 ^abc^	878 ± 78 ^c^
2	295 ± 10 ^f^	1.5 ± 0.04 ^n^	2428 ± 118 ^a^	33.1 ± 1.2 ^efg^	0.66 ± 0.01 ^bc^	94.8 ± 2.1 ^bcdefg^	1530 ± 53 ^a^
3	317 ± 5 ^e^	2.8 ± 0.05 ^h^	975 ± 47 ^e^	27.2 ± 0.5 ^k^	0.65 ± 0.01 ^bc^	88.1 ± 1.1 ^ijk^	578 ± 10 ^e^
4	353 ± 5 ^c^	2.9 ± 0.01 ^gh^	734 ± 26 ^gh^	29.1 ± 1.7 ^ijk^	0.61 ± 0.00 ^fgh^	93.8 ± 3.4 ^defghi^	367 ± 17 ^hij^
5	356 ± 7 ^c^	3.0 ± 0.08 ^fg^	670 ± 15 ^hijk^	29.1 ± 0.9 ^ijk^	0.56 ± 0.01 ^ij^	89.6 ± 2.4 ^ghijk^	399 ± 14 ^gh^
6	343 ± 3 ^cd^	2.3 ± 0.04 ^jk^	940 ± 47 ^e^	32.1 ± 1.1 ^fg^	0.63 ± 0.02 ^cdef^	90.0 ± 2.1 ^ghijk^	561 ± 0 ^e^
7	330 ± 8 ^de^	3.0 ± 0.07 ^fg^	604 ± 44 ^klm^	23.7 ± 0.8 ^l^	0.59 ± 0.01 ^hi^	85.3 ± 1.4 ^k^	317 ± 12 ^jk^
8	259 ± 11 ^ik^	3.5 ± 0.09 ^cd^	601 ± 15 ^klm^	28.8 ± 0.5 ^ijk^	0.70 ± 0.02 ^a^	100.7 ± 1.5 ^ab^	391 ± 43 ^hi^
9	279 ± 5 ^g^	3.8 ± 0.12 ^b^	537 ± 38 ^mno^	34.3 ± 0.2 ^cde^	0.72 ± 0.01 ^a^	98.7 ± 4.2 ^abcde^	365 ± 8 ^hij^
10	330 ± 3 ^de^	1.8 ± 0.02 ^m^	1618 ± 41 ^b^	30.3 ± 1.0 ^hi^	0.65 ± 0.02 ^bc^	93.0 ± 7.2 ^efghi^	1007 ± 55 ^b^
11	325 ± 1 ^e^	3.0 ± 0.03 ^fg^	525 ± 37 ^mno^	27.7 ± 0.5 ^k^	0.64 ± 0.00 ^bcde^	92.0 ± 4.6 ^fghij^	343 ± 10 ^hijk^
12	384 ± 14 ^b^	3.0 ± 0.09 ^fg^	679 ± 44 ^hijk^	27.3 ± 1.2 ^k^	0.59 ± 0.01 ^hi^	85.0 ± 2.8 ^k^	343 ± 22 ^hijk^
13	354 ± 10 ^c^	2.5 ± 0.06 ^i^	842 ± 29 ^f^	29.64± 1.3 ^ij^	0.62 ± 0.02 ^defg^	88.1 ± 0.9 ^ijk^	459 ± 2 ^fg^
14	319 ± 14 ^e^	3.3 ± 0.05 ^e^	623 ± 19 ^jkl^	23.8 ± 0.4 ^l^	0.63 ± 0.02 ^cdef^	84.6 ± 2.9 ^k^	320 ± 20 ^ijk^
15	303 ± 9 ^f^	3.5 ± 0.05 ^d^	801 ± 36 ^fg^	33.6 ± 0.5 ^def^	0.62 ± 0.02 ^defg^	98.2 ± 5.6 ^abcde^	478 ± 17 ^f^
16	247 ± 3 ^jk^	2.0 ± 0.02 ^l^	1258 ± 49 ^c^	36.4 ± 0.7 ^ab^	0.66 ± 0.01 ^b^	95.9 ± 1.6 ^abcdef^	815 ± 1 ^d^
17	281 ± 6 ^g^	3.8 ± 0.05 ^b^	400 ± 16 ^p^	22.0 ± 0.5 ^m^	0.53 ± 0.02 ^k^	87.0 ± 4.4 ^jk^	208 ± 22 ^l^
18	202 ± 9 ^o^	3.1 ± 0.12 ^f^	829 ± 35 ^f^	35.7 ± 0.6 ^bc^	0.71 ± 0.01 ^a^	95.4 ± 1.5 ^bcdefg^	572 ± 7 ^e^
19	187 ± 12 ^p^	3.2 ± 0.02 ^e^	716 ± 32 ^hi^	35.2 ± 1.2 ^bcd^	0.70 ± 0.01 ^a^	94.7 ± 2.1 ^cdefgh^	470 ± 18 ^f^
20	230 ± 5 ^lm^	2.9 ± 0.07 ^h^	638 ± 30 ^ijkl^	28.4 ± 1.5 ^jk^	0.64 ± 0.02 ^bcdef^	89.0 ± 3.7 ^hijk^	373 ± 7 ^hij^
21	273 ± 2 ^gh^	3.5 ± 0.06 ^d^	510 ± 17 ^no^	29.5 ± 0.2 ^ij^	0.61 ± 0.02 ^efgh^	98.1 ± 2.7 ^abcde^	286 ± 2 ^k^
22	216 ± 4 ^mn^	3.6 ± 0.08 ^c^	452 ± 38 ^op^	37.5 ± 1.6 ^a^	0.66 ± 0.02 ^bc^	100.5 ± 1.2 ^abc^	281 ± 25 ^k^
23	222 ± 7 ^mn^	2.8 ± 0.05 ^h^	515 ± 30 ^no^	31.7 ± 0.7 ^gh^	0.64 ± 0.02 ^bcd^	101.7 ± 1.5 ^a^	340 ± 4 ^hijk^
24	211 ± 8 ^no^	2.2 ± 0.06 ^k^	573 ± 27 ^lmn^	32.6 ± 1.0 ^fg^	0.59 ± 0.02 ^gh^	97.0 ± 3.4 ^abcdef^	335 ± 41 ^hijk^
25	215 ± 9 ^no^	2.8 ± 0.05 ^h^	506 ± 34 ^no^	36.9 ± 2.3 ^ab^	0.64 ± 0.01 ^bcd^	95.3 ± 1.6 ^bcdefg^	325 ± 4 ^ijk^
26	453 ± 11 ^a^	2.9 ± 0.09 ^gh^	621 ± 43 ^jkl^	16.4 ± 0.5 ^n^	0.56 ± 0.02 ^j^	79.1 ± 3.1 ^l^	276 ± 11 ^k^
27	153 ± 1 ^q^	4.2 ± 0.10 ^a^	477 ± 22 ^op^	31.9 ± 0.6 ^gh^	0.72 ± 0.01 ^a^	99.0 ± 1.9 ^abcd^	332 ± 16 ^hijk^
28	264 ± 3 ^hi^	2.8 ± 0.03 ^h^	695 ± 61 ^hij^	28.3 ± 0.6 ^jk^	0.65 ± 0.01 ^bc^	93.6 ± 1.9 ^defghi^	464 ± 64 ^f^
Ave. soy	320 ± 41 a	2.8 ± 0.6 a	917 ± 522 a	29.5 ± 3.6 b	0.64 ± 0.05 a	91.7 ± 5.5 b	561 ± 350 a
Ave. pea	246 ± 43 b	3.1 ± 0.6 a	736 ± 277 a	31.5 ± 5.2 ab	0.64 ± 0.06 a	94.0 ± 4.4 ab	457 ± 200 a
Ave. wheat	216 ± 5 b	2.9 ± 0.6 a	512 ± 49.55 a	34.7 ± 3.0 a	0.63 ± 0.03 a	98.6 ± 3.0 a	320 ± 27 a
Average ^B^	284	2.9	780	30.3	0.64	93.1	479
LSD (5%) ^C^	13	0.1	74	1.6	0.02	5.0	61

Abbreviations: RHC, rehydration capacity of TVP. ^A^ Protein types of samples: 1–14 (soy protein), 15–21 (pea protein), 22–25 (wheat gluten), 26 (chickpea protein), 27 (pea and chickpea protein mixture), 28 (pea and navy bean protein mixture). Means with different superscript letters within the same column are significantly different (*p* < 0.05) among samples 1–28. Different lowercase letters indicate significant difference among means of soy, pea and wheat gluten samples within the same column (*p* < 0.05). ^B,C^ Average values of all samples and least significant difference (LSD) for comparison of different samples.

**Table 5:** Cooking properties of TVP-based patties.

Sample ^A^	Cooking Loss (%)	Diameter Shrinkage (%)	Moisture Retention (%)	Fat Retention (%)
1	14.7 ± 0.8 ^ij^	6.4 ± 0.5 ^jkl^	78.2 ± 0.6 ^bcd^	89.0 ± 0.8 ^b^
2	11.6 ± 0.6 ^m^	4.4 ± 0.5 ^n^	78.0 ± 1.1 ^bcde^	84.4 ± 0.4 ^cd^
3	14.8 ± 0.5 ^hij^	7.3 ± 0.5 ^fghi^	75.7 ± 0.0 ^ghi^	83.1 ± 1.2 ^de^
4	17.0 ± 0.6 ^bc^	8.7 ± 0.9 ^bc^	76.2 ± 0.7 ^efghi^	80.0 ± 1.0 ^gh^
5	16.7 ± 0.9 ^c^	7.7 ± 0.6 ^defgh^	74.8 ± 0.8 ^ij^	79.9 ± 1.8 ^gh^
6	14.9 ± 0.8 ^hij^	6.9 ± 0.4 ^hij^	73.6 ± 0.8 ^j^	81.0 ± 0.8 ^fg^
7	15.2 ± 0.5 ^fghi^	7.7 ± 0.6 ^defgh^	77.6 ± 1.3 ^cdefg^	79.8 ± 1.0 ^gh^
8	17.4 ± 0.4 ^b^	9.0 ± 0.7 ^ab^	75.5 ± 0.6 ^hi^	79.4 ± 1.0 ^gh^
9	16.4 ± 1.0 ^cd^	7.4 ± 0.7 ^efgh^	75.9 ± 1.5 ^fghi^	80.9 ± 0.7 ^fg^
10	12.3 ± 0.6 ^l^	4.6 ± 0.3 ^n^	78.1 ± 0.7 ^bcde^	84.9 ± 1.4 ^c^
11	13.7 ± 0.6 ^k^	6.6 ± 0.5 ^ijk^	77.8 ± 0.7 ^cdef^	85.5 ± 0.4 ^c^
12	15.5 ± 0.5 ^efg^	7.8 ± 0.9 ^defg^	76.3 ± 0.3 ^defghi^	77.4 ± 1.5 ^ij^
13	16.0 ± 0.6 ^de^	8.2 ± 0.6 ^bcde^	76.6 ± 1.6 ^defghi^	80.6 ± 0.3 ^fg^
14	15.9 ± 0.6 ^def^	8.3 ± 0.7 ^bcd^	78.0 ± 0.8 ^cde^	78.8 ± 0.5 ^hi^
15	18.5 ± 1.0 ^a^	8.2 ± 0.7 ^bcde^	73.2 ± 1.1 ^j^	70.5 ± 0.4 ^l^
16	11.6 ± 0.8 ^m^	4.6 ± 0.4 ^n^	77.7 ± 1.8 ^cdef^	92.4 ± 0.4 ^a^
17	15.6 ± 0.6 ^efg^	9.5 ± 0.7 ^a^	77.1 ± 0.7 ^defgh^	80.0 ± 0.5 ^gh^
18	13.7 ± 0.9 ^k^	6.5 ± 0.8 ^ijkl^	77.8 ± 1.1 ^cdef^	85.1 ± 1.1 ^c^
19	14.3 ± 0.7 ^jk^	6.1 ± 0.5 ^klm^	77.0 ± 1.1 ^defgh^	82.8 ± 0.7 ^e^
20	14.8 ± 0.7 ^hij^	8.0 ± 0.7 ^cdef^	76.0 ± 0.2 ^fghi^	76.0 ± 0.5 ^j^
21	15.9 ± 0.7 ^def^	5.6 ± 0.3 ^m^	75.4 ± 0.3 ^hi^	74.3 ± 0.2 ^k^
22	15.1 ± 0.5 ^ghi^	7.7 ± 0.7 ^defgh^	77.9 ± 1.0 ^cde^	81.0 ± 0.5 ^fg^
23	15.2 ± 0.4 ^ghi^	7.9 ± 0.6 ^cdef^	80.3 ± 0.3 ^a^	81.8 ± 0.4 ^ef^
24	12.2 ± 0.6 ^lm^	7.0 ± 0.5 ^ghij^	79.1 ± 0.3 ^abc^	79.5 ± 0.6 ^gh^
25	13.7 ± 0.7 ^k^	5.7 ± 0.8 ^lm^	77.7 ± 1.1 ^cdef^	80.5 ± 1.3 ^fg^
26	12.3 ± 0.3 ^l^	5.9 ± 0.5 ^klm^	80.5 ± 0.4 ^a^	91.5 ± 0.6 ^a^
27	15.8 ± 1.1 ^defg^	8.2 ± 0.7 ^bcd^	76.3 ± 0.9 ^defghi^	82.7 ± 0.6 ^e^
28	13.6 ± 0.5 ^k^	6.5 ± 0.6 ^ijkl^	79.8 ± 1.8 ^ab^	81.1 ± 0.7 ^fg^
Ave. soy	15.2 ± 1.7 a	7.2 ± 1.4 a	76.6 ± 1.4 b	81.8 ± 3.2 a
Ave. pea	14.9 ± 2.1 a	6.9 ± 1.7 a	76.3 ± 1.6 b	80.2 ± 7.4 a
Ave. wheat	14.0 ± 1.4 a	7.1 ± 1.0 a	78.8 ± 1.2 a	80.7 ± 1.0 a
Average ^B^	14.8	7.1	77.1	81.6
LSD (5%) ^C^	0.6	0.7	1.6	1.5

^A^ Protein types of samples: 1–14 (soy protein), 15–21 (pea protein), 22–25 (wheat gluten), 26 (chickpea protein), 27 (pea and chickpea protein mixture), 28 (pea and navy bean protein mixture). Means with different superscript letters within the same column are significantly different (*p* < 0.05) among samples 1–28. Different lowercase letters indicate significant difference among means of soy, pea and wheat gluten samples within the same column (*p* < 0.05). ^B,C^ Average values of all samples and least significant difference (LSD) for comparison of different samples.

**Table 6:** Textural properties of TVP-based patties.

Sample ^A^	Hardness (g)	Resilience (%)	Cohesiveness	Springiness (%)	Chewiness (g)	Shear Force (g)	Compressed Juiciness (%)
1	1554 ± 12 ^e^	4.7 ± 0.1 ^hijk^	0.20 ± 0.00 ^ijk^	55.4 ± 1.2 ^kl^	173 ± 7 ^h^	432 ± 47 ^cd^	7.1 ± 0.7 ^hi^
2	2768 ± 47 ^a^	5.3 ± 0.2 ^fg^	0.20 ± 0.01 ^jk^	50.1 ± 0.3 ^m^	270 ± 12 ^d^	527 ± 63 ^b^	4.4 ± 0.5 ^k^
3	965 ± 21 ^ijkl^	4.1 ± 0.3 ^lm^	0.20 ± 0.02 ^jk^	53.2 ± 3.1 ^lm^	95 ± 9 ^kl^	255 ± 13 ^ijkl^	8.9 ± 0.5 ^cde^
4	1055 ± 60 ^h^	4.6 ± 0.1 ^jk^	0.20 ± 0.01 ^ijk^	56.8 ± 0.9 ^jkl^	134 ± 14 ^ijk^	151 ± 23 ^mn^	7.7 ± 0.4 ^gh^
5	1249 ± 16 ^g^	4.7 ± 0.2 ^hijk^	0.20 ± 0.01 ^jk^	56.2 ± 2.5 ^kl^	136 ± 6 ^ijk^	325 ± 34 ^fg^	7.2 ± 0.5 ^ghi^
6	1653 ± 139 ^d^	5.0 ± 0.4 ^gh^	0.21 ± 0.00 ^hij^	57.6 ± 2.2 ^jk^	197 ± 15 ^fg^	441 ± 14 ^cd^	5.8 ± 0.5 ^j^
7	878 ± 6 ^lm^	4.3 ± 0.3 ^klm^	0.23 ± 0.00 ^fgh^	67.9 ± 1.9 ^efg^	141 ± 7 ^ij^	354 ± 43 ^f^	8.4 ± 0.4 ^ef^
8	681 ± 18 ^n^	5.0 ± 0.3 ^ghi^	0.28 ± 0.01 ^e^	69.4 ± 1.1 ^ef^	130 ± 7 ^jk^	318 ± 24 ^fgh^	10.3 ± 0.5 ^b^
9	1011 ± 78 ^hij^	5.0 ± 0.2 ^ghij^	0.28 ± 0.02 ^e^	67.4 ± 2.3 ^fg^	176 ± 15 ^h^	285 ± 45 ^ghi^	9.0 ± 0.4 ^cde^
10	2457 ± 55 ^c^	5.4 ± 0.4 ^f^	0.20 ± 0.01 ^ijk^	53.5 ± 2.7 ^lm^	260 ± 11 ^de^	809 ± 93 ^a^	4.4 ± 0.5 ^k^
11	929 ± 34 ^jkl^	4.7 ± 0.2 ^hijk^	0.22 ± 0.01 ^ghi^	60.2 ± 1.6 ^ij^	122 ± 5 ^jk^	213 ± 45 ^kl^	7.7 ± 0.7 ^gh^
12	1020 ± 32 ^hi^	4.6 ± 0.3 ^ijk^	0.20 ± 0.01 ^jk^	55.6 ± 2.3 ^kl^	113 ± 6 ^kl^	203 ± 36 ^lm^	7.1 ± 0.5 ^hi^
13	1388 ± 59 ^f^	4.4 ± 0.2 ^kl^	0.19 ± 0.00 ^k^	50.9 ± 0.6 ^m^	127 ± 5 ^jk^	369 ± 28 ^ef^	7.4 ± 0.3 ^gh^
14	803 ± 27 ^m^	4.5 ± 0.2 ^kl^	0.24 ± 0.00 ^fg^	62.1 ± 2.8 ^hi^	125 ± 5 ^jk^	230 ± 32 ^ijkl^	10.3 ± 0.4 ^b^
15	799 ± 21 ^m^	4.5 ± 0.1 ^kl^	0.20 ± 0.02 ^ijk^	62.1 ± 1.3 ^hi^	93 ± 5 ^kl^	131 ± 20 ^n^	8.9 ± 0.4 ^cde^
16	2542 ± 42 ^b^	11.5 ± 0.6 ^a^	0.22 ± 0.01 ^ghij^	71.3 ± 2.1 ^de^	391 ± 9 ^a^	415 ± 52 ^de^	4.0 ± 0.2 ^k^
17	559 ± 27 ^o^	5.2 ± 0.3 ^fg^	0.31 ± 0.03 ^cd^	73.0 ± 0.7 ^cd^	134 ± 12 ^ijk^	266 ± 11 ^hijk^	12.9 ± 1.0 ^a^
18	1598 ± 85 ^de^	8.2 ± 0.2 ^c^	0.29 ± 0.01 ^de^	65.0 ± 0.7 ^gh^	308 ± 11 ^b^	449 ± 10 ^cd^	6.6 ± 0.5 ^i^
19	1430 ± 67 ^f^	7.1 ± 0.3 ^d^	0.27 ± 0.02 ^e^	65.5 ± 1.6 ^gh^	294 ± 30 ^bc^	490 ± 32 ^bc^	6.7 ± 0.7 ^i^
20	913 ± 51 ^kl^	4.0 ± 0.1 ^m^	0.20 ± 0.01 ^ijk^	64.5 ± 1.7 ^gh^	116 ± 6 ^k^	235 ± 24 ^ijkl^	7.6 ± 0.4 ^gh^
21	596 ± 32 ^o^	5.5 ± 0.2 ^f^	0.38 ± 0.01 ^a^	90.6 ± 0.5 ^a^	193 ± 10 ^gh^	69 ± 4 ^o^	10.1 ± 0.5 ^b^
22	887 ± 23 ^l^	9.0 ± 0.5 ^b^	0.39 ± 0.02 ^a^	75.1 ± 1.2 ^c^	275 ± 3 ^cd^	212 ± 19 ^kl^	9.2 ± 0.9 ^cd^
23	887 ± 16 ^l^	6.3 ± 0.3 ^e^	0.32 ± 0.01 ^bc^	75.6 ± 1.8 ^c^	216 ± 4 ^f^	224 ± 24 ^jkl^	9.3 ± 1.0 ^c^
24	1048 ± 46 ^hi^	7.1 ± 0.1 ^d^	0.34 ± 0.01 ^b^	75.6 ± 1.6 ^c^	271 ± 29 ^d^	451 ± 19 ^cd^	7.7 ± 0.7 ^gh^
25	978 ± 71 ^hijk^	7.2 ± 0.1 ^d^	0.32 ± 0.01 ^bc^	82.6 ± 1.3 ^b^	245 ± 9 ^e^	277 ± 17 ^ghij^	7.9 ± 0.5 ^fg^
26	891 ± 49 ^l^	4.5 ± 0.2 ^kl^	0.23 ± 0.01 ^fgh^	79.9 ± 2.4 ^b^	153 ± 19 ^i^	151 ± 16 ^mn^	8.5 ± 0.3 ^de^
27	801 ± 11 ^m^	5.2 ± 0.1 ^fg^	0.25 ± 0.01 ^f^	66.9 ± 3.1 ^fg^	135 ± 11 ^ijk^	264 ± 22 ^hijk^	10.1 ± 0.4 ^b^
28	964 ± 22 ^ijkl^	4.5 ± 0.3 ^kl^	0.20 ± 0.01 ^ijk^	58.1 ± 2.8 ^jk^	117 ± 4 ^k^	264 ± 18 ^hijk^	7.8 ± 0.6 ^fg^
Ave. soy	1315 ± 618 a	4.7 ± 0.4 b	0.22 ± 0.03 b	58.3 ± 6.3 b	157 ± 53 b	351 ± 168 a	7.5 ± 1.8 a
Ave. pea	1205 ± 711 a	6.6 ± 2.6 a	0.27 ± 0.06 b	70.3 ± 9.8 a	218 ± 114 ab	294 ± 163 a	8.1 ± 2.8 a
Ave. wheat	950 ± 78 a	7.4 ± 1.1 a	0.34 ± 0.03 a	77.2 ± 3.6 a	252 ± 28 a	291 ± 11 a	8.5 ± 0.9 a
Average ^B^	1189	5.6	0.25	65.1	184	315	8.0
LSD (5%) ^C^	75	0.4	0.02	3.2	20	52	0.6

^A^ Protein types of samples: 1–14 (soy protein), 15–21 (pea protein), 22–25 (wheat gluten), 26 (chickpea protein), 27 (pea and chickpea protein mixture), 28 (pea and navy bean protein mixture). Means with different superscript letters within the same column are significantly different (*p* < 0.05) among samples 1–28. Different lowercase letters indicate significant difference among means of soy, pea and wheat gluten samples within the same column (*p* < 0.05). ^B,C^ Average values of all samples and least significant difference (LSD) for comparison of different samples.

## Data Availability

The data that support the findings of this study are included in the paper.
